# Etiology of Renal Replacement Therapy in Iran

**DOI:** 10.1155/2019/5010293

**Published:** 2019-11-26

**Authors:** Negar Morovatdar, Gholamreza Tayebi Nasrabad, Konstantinos Tsarouhas, Ramin Rezaee

**Affiliations:** ^1^Clinical Research Unit, Imam Reza Hospital, Faculty of Medicine, Mashhad University of Medical Sciences, Mashhad, Iran; ^2^Treatment Affair of Vice Chancellery, Mashhad University of Medical Sciences, Mashhad, Iran; ^3^Department of Cardiology, University Hospital of Larissa, Larissa, Greece; ^4^Center for Adolescent Medicine and UNESCO Chair on Adolescent Health Care, First Department of Pediatrics, Medical School, National and Kapodistrian University of Athens, Aghia Sophia Children's Hospital, Athens, Greece; ^5^Department of Chemical Engineering, Environmental Engineering Laboratory, Aristotle University of Thessaloniki, Thessaloniki, Greece

## Abstract

**Introduction:**

End-stage renal disease (ESRD) is one of the most common life-threatening diseases. In the past two decades, several factors were held responsible as the cause of this condition. The present study aimed to determine the causes of ESRD in the province of Khorasan Razavi, Iran.

**Materials and Methods:**

This cross-sectional study was conducted on 2404 ESRD patients who referred to 39 hemodialysis centers in Khorasan Razavi province, Iran, and were registered in the Mashhad University of Medical Sciences (MUMS), between 2000 and December 2018. Sociodemographic data and causes of ESRD were extracted from data registry.

**Results:**

The mean age at onset of hemodialysis for 2404 patients was 52.8 ± 16.4 years, and 57.1% of the patients were male. Clinical profile of hypertension (28.3%) and diabetes mellitus (24.8%) were the most common known causes of ESRD in our patients. Hypertension was more prevalent in male patients compared with females (30 vs 25%, respectively) while diabetes was more prevalent in females compared with males (25.4 vs 24.4%, respectively), *p*=0.009. Educational level was significantly associated with the cause of ESRD (*p* < 0.001). Age of onset of ESRD in hypertensive patients was significantly lower compared with diabetic patients (51.5 ± 16.3 vs 58.28 ± 12.9 years, respectively; *p* < 0.001).

**Conclusions:**

In the current study, the most common causes of ESRD were hypertension and diabetes mellitus. Primary prevention of hypertension and diabetes and proper treatment must be considered to reduce the burden of ESRD in Iran.

## 1. Introduction

End-stage renal disease (ESRD) is one of the most prevalent life-threatening diseases. It is estimated that 2000 individuals per million in Japan, about 1500 per million in the United States, and about 800 per million in the European Union are suffering from ESRD, and these numbers continue to increase in most countries [1]. Currently, it is estimated that more than 24,000 people with ESRD live in Iran, and their population has drastically increased over the recent years [2]. The disability-adjusted life years (DALY) for ESRD and chronic kidney disease (CKD) were 21,490 and 1, 124, 164 years, respectively, in Iran [2]. The prevalence and incidence of ESRD increased in Iran from, respectively, 137 and 13.82 per million in 1997, to 238 and 49.9 per million in 2000, and 357 and 63.8 per million people in 2006 [3]. Different methods such as hemodialysis/peritoneal dialysis and kidney transplantation are employed as renal replacement therapies. The most common renal replacement therapy in Iran is hemodialysis, and the prevalence and incidence rates of ESRD patients undergoing hemodialysis increased from 98 and 38.2 per million per year, to 169 and 66 per million people per year, respectively, in 2004 [2, 4]. Currently, diabetes is considered the major cause of end-stage renal failure in most countries [5]. Data from the Hong Kong Renal Registry also showed a progressive increase in the number of diabetics requiring dialysis, accounting now for 38% of the patients, while only 23% had glomerulonephritis. Other Asian countries also have high percentages of end-stage renal failure patients due to diabetes (e.g., 42% in Pakistan, 35% in Taiwan, 25% in Philippines, and 37% in Japan) [5]. Many studies reported the epidemiological aspects as well as the cause of ESRD in different developed countries, but few studies were conducted in developing countries. The aim of this study was to present the epidemiological pattern of ESRD patients undergoing maintenance hemodialysis and the most common causes of this morbidity in Khorasan Razavi province of Iran. Based on the last census conducted in 2016 in Iran [6], the total population of Khorasan Razavi province is 6,434,501 inhabitants, including 49.5% females and 50.4% males. Khorasan Razavi province is ranked second in the country after Tehran province, accounting for 8.05% of the total Iranian population.

## 2. Methods

This cross-sectional epidemiological study was conducted on 2404 ESRD patients who referred to 39 hemodialysis centers in Khorasan Razavi province, northeast of Iran, from 2000 to December 2018. The study was carried out upon the approval of Mashhad University of Medical Sciences (registration no. 921285). In this study, the definition used for ESRD was “*permanent and irreversible loss of kidney function requiring renal replacement therapy*.” We only included hemodialysis patients who had been on dialysis up to December 2018. Patients who died during the study period were excluded. Other exclusion criteria were incomplete data, hemodialysis because of acute kidney failure, and kidney transplantation or peritoneal dialysis employed as renal replacement therapy at any time during the study period. The patients were divided into 3 groups based on their age (i.e., <40 years, 40–70 years, and >70 years). Data are presented as mean ± standard deviation for continuous variables and as frequencies (percentages) for categorical variables. The SPSS software (Statistical Package for the Social Sciences, version 16.0, SPSS Inc., Chicago, Ill, USA) was used for data analysis. To compare continuous variables, Mann–Whitney and Kruskal–Wallis tests were used. Categorical variables were compared by the chi-square test. The level of significance was set at <0.05.

## 3. Results

**Figure 1:**
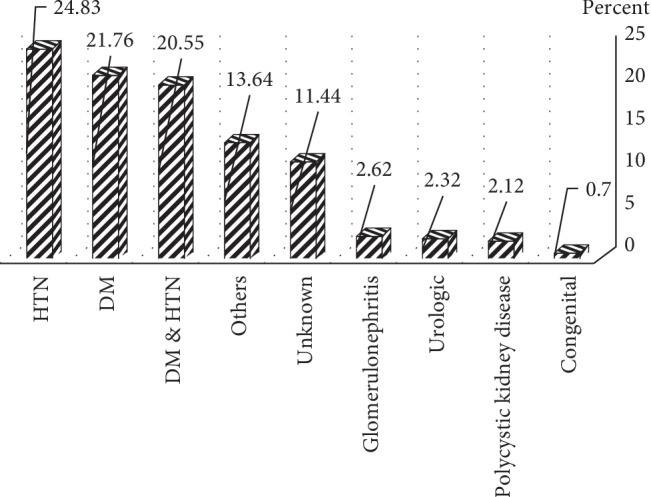
Frequencies of end-stage renal disease (ESRD) causing in hemodialysis patients.

**Figure 2:**
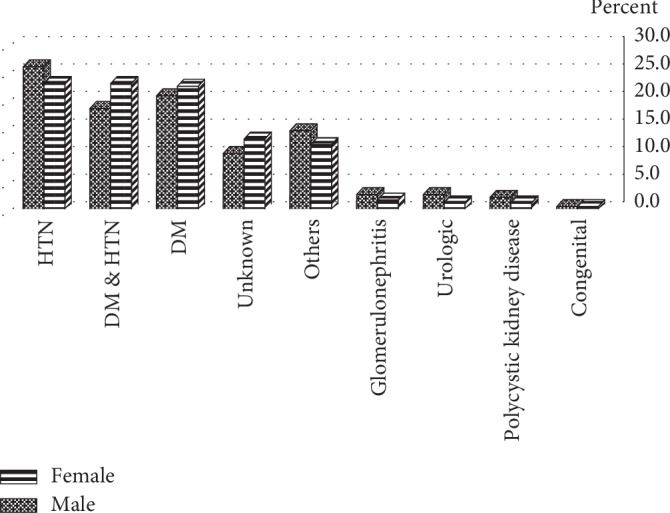
Sex-related frequencies of end-stage renal disease (ESRD) causing in hemodialysis patients.

**Figure 3:**
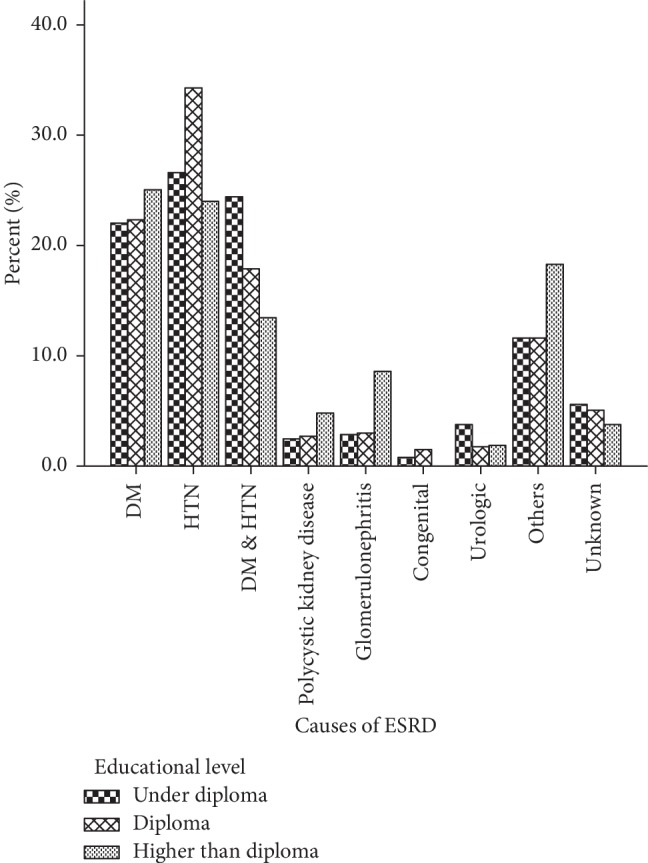
Causes of end-stage renal disease (ESRD) in hemodialysis patients with regards to different educational levels.

**Figure 4:**
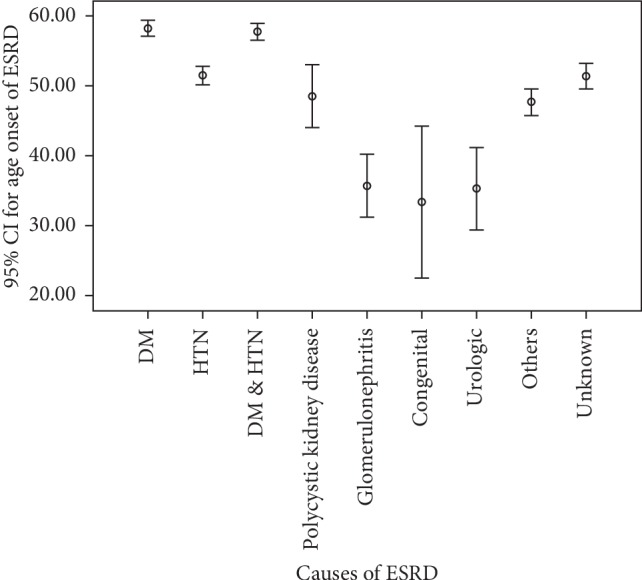
Age onset of ESRD according to disease's causality.

The mean age of onset of ERSD in 2404 patients included in this study was 52.8 ± 16.4 years. In the study population, 1372 (57.1%) subjects were male, and the male/female ratio was 1.3. Concerning the educational status of the study population, 45% did not have a high school diploma, 27% were illiterate, 21% had high school diploma, and only 6.5% had higher education. Moreover, 2196 (91.3%) of patients received haemodialysis three times a week, 7.4% received it two times a week, and 1.3% received it once a week. Clinical profile of hypertension (Hypertension) (28.3%), diabetes mellitus (24.8%), and combined hypertension-diabetes mellitus (23.4%) were the most common causes of ESRD in the study population. Other causes of ESRD were polycystic kidney disease (2.4%), glomerulonephritis (2.6%), and congenital disease (0.8%) (Figure 1). We did not find the cause of ESRD in 275 (13.1%) of our patients. In our study, 538 patients (22.4%) were <40 years old, 1543 (64.2%) were 40–70 years, and 323 (13.4%) were >70 years old. The cause of ESRD significantly varied among different age groups; hypertension in patients under 40 years of age and diabetes in patients over 40 years were the most common cause of ESRD (*p* < 0.001 for both cases). We found that the cause of ESRD significantly varied between male and female patients; hypertension was the dominant cause in male population (30 vs 25%) while diabetes was more prevalent in females (25.4 vs 24.4%; *p*=0.009) (Figure 2). Educational level was significantly associated with the cause of ESRD, as hypertension was more prevalent in patients with high school diploma or lower education, compared with patients with higher education among whom, diabetes was the most common cause of ESRD (37.8 vs 28.7%, *p* < 0.001 (Figure 3). We did not find any association between blood type and cause of ESRD in this population (*p*=0.06). Age of onset of ESRD significantly differed among various causes; in this regard, age of onset of ESRD in hypertensive patients was significantly lower than that of diabetic patients (51.5 ± 16.3 vs 58.28 ± 12.9 years, respectively; *p* < 0.001) (Figure 4).

## 4. Discussion

In this cross-sectional study, we found that hypertension and diabetes mellitus are the most common causes of ESRD in the northeast of Iran. Also, the causes of ESRD were significantly associated with age, sex, and educational level as hypertension was more prevalent in male patients with education level lower than high school diploma.

Our results were consistent with other studies conducted in Iran [7], but in contrast to data published by Salahi et al. [8], who reported glomerulonephritis (GN) and hypertension as the most common causes of ESRD. This variation may be due to increase in lifestyle-dependent vascular risk factors such as obesity, diabetes, and hypertension in developing countries [9]. Monfared et al. showed that the most common causes of ESRD in Guilan, Iran were hypertension, unknown cause, and diabetes mellitus [10]. Studies conducted in developed countries showed different results as they found renal vascular disease and diabetic nephropathy as the leading causes of ESRD [11]. Hypertensive nephropathy was the most common cause of ESRD in a study conducted in Switzerland [12], while in the ERA-EDTA registry, the prevalence of hypertension as a cause of ESRD requiring renal replacement therapy was quite lower, reaching 14.7% for the years 1998-99 [13]. In the Turkish version of the DOPPS survey, very similar patterns of hypertension as the primary cause of ESRD were found accounting for 32% of the cases, while the Europe DOPPS registry presented hypertension as ESRD etiology only in 19% of the patients [14]. At the same time, DOPPS registry data for Japan presented hypertension as the primary cause of ESRD in only 6% of the patients with glomerulonephritis/vasculitis rising to 40%. It is possible that late presentation of patients in the renal disease continuum in medical facilities in developing countries may account in part for these differences rendering renal biopsies inconclusives. Dharan et al. on the other hand showed that GN was the most common cause of ESRD in India [15]. Diabetic nephropathy is a primary cause of ESRD with significant heterogeneity across geographic regions and continents. In Japan, the J-DOPPS registry recorded an impressive 35% of patients with diabetes as ESRD cause, while in Europe, diabetes accounted for 25% and in North America for 43% of the cases. In the current registry, diabetes' causality of ESRD disease was comparable to Japan and North America taking in mind 24.8% of diabetes mellitus and 23.4% of combined hypertension-diabetes mellitus as a primary cause of ESRD in Iran. The ERA-EDTA registry in Europe and the USRDS (US renal data system) in USA revealed great differences in the diabetic ESRD population between the two continents that undergo renal replacement therapy (RRT); in Europe, diabetic patients undergoing RRT are 35% less than those in the USA, having reached 94.8 pmp in 2000 [13, 16]. In the above-mentioned studies, an unknown cause of ESRD was recorded at 18% [17] and 14% [7] of cases, which was similar to our findings, where in 13.1% of the patients, the cause of ESRD was unknown. High percentage of unknown cause is a concern for the improvement of pre-ESRD workup. The said high rates have also been reported in developing countries [18, 19], which could be due to late referral and diagnosis of ESRD.

In our study, the ratio of male to female was 1.3 with dominancy of the male population. This result was consistent with data reported by other studies conducted in Iran [10, 20] as well as studies in developed countries [21].

Considering the different age categories, hypertension in patients under 40 years old and diabetes in those above 40 years were the most common causes of ESRD. In another study conducted in Guilan, Iran, the most common causes of ESRD were diabetes mellitus, hypertension, and an unknown cause in patients aged ≥60 years, but urinary calculi, unknown cause, and polycystic kidney disease were the main causes of ESRD in patients aged 30–44 years [10]. In Fars province of Iran, the most common causes of ESRD in subjects aged under 40 years were unknown cause and hypertension, while diabetes mellitus was the main cause of ESRD in patients ≥40 years [7]. We found that hypertension was more prevalent in male patients while diabetes was more prevalent in female patients; however, in another study done in Iran, for both sexes, hypertension was the most common cause of ESRD [10].

We found that educational level was significantly associated with the cause of ESRD, as hypertension was more prevalent in patients with lower educational level, while diabetes mellitus was more prevalent in subjects with higher educational level. This finding might be due to socioeconomic-dependent factors.

To the best of our knowledge, this study is the first in northeast Iran concerning the epidemiological pattern of ESRD causes in patients who underwent hemodialysis. In the registry of ESRD patients of the current study, data regarding renal biopsies in the said patients were not recorded even when performed. Renal biopsies are recommended to be used in the Iranian healthcare system in the diagnostic workup of ESRD patients. Unfortunately, in the countryside of Iran as in other developing countries, patients present themselves to the ED at an advanced clinical state, and therefore, biopsies are applied in a lesser extent due to emerging treatment requirements. At this stage, the rate of renal biopsy utilization in the diagnostic workup of ESRD patients in the current registry cannot be safely described. There is a great need for a broader implementation of this diagnostic tool.

## 5. Conclusion

In our study, the most common causes of ESRD were hypertension and diabetes mellitus. Primary prevention of hypertension and diabetes and secondary prevention with proper and timely treatment of these two risk factors must be considered to reduce the burden of ESRD in Iran.

## Data Availability

The SPSS file of data used to support the findings of this study were supplied by Mashhad University of Medical Sciences under license and so cannot be made freely available.
